# UNDERSTANDING HEALTHCARE UTILIZATION AND COGNITION IN OLDER LATINO/A ADULTS: A COMMUNITY-BASED APPROACH

**DOI:** 10.1093/geroni/igae098.2261

**Published:** 2024-12-31

**Authors:** Sayeem Hossain, Adriana Perez

**Affiliations:** University of Pennsylvania, Philadelphia, Pennsylvania, United States; University of Pennsylvania, Philadelphia, Pennsylvania, United States

## Abstract

Cognitive decline and Alzheimer’s Disease present significant public health challenges, particularly for older Latino/a adults who experience high risk of cognitive impairment. The Tiempo Juntos por Nuestra Salud study tests a culturally relevant, community-based clinical trial designed to enhance physical activity, cardiovascular health, sleep quality, and cognitive function in older Latino/a adults with mild cognitive impairment (MCI). The data was obtained from the Tiempo Juntos participant dataset including 236 Spanish-speaking Latino/as aged 55 and older with MCI in North Philadelphia. Participant scores on cognition assessments and various survey instruments were used to conduct analyses. This secondary analysis examines 1) socioeconomic predictors of cognitive function, education level and monthly income levels in comparison to cognition scores in an analysis of variance; 2) predictors of healthcare utilization, using multiple regression to compare the number of clinician visits with variables related to neighborhood walkability, self-rated motivation, self-rated frequency of physical activity, and functional assessment score. Results showed that education and monthly income were significant socioeconomic drivers in later-life cognition. Healthcare utilization was negatively associated with neighborhood walkability but positively associated with motivation. Healthcare utilization is likely reduced by having environments conducive to doing physical activity. Greater individual motivation to improve health can lead to more connection to health services. This study highlights the impact of socioeconomic factors on health outcomes in a culturally tailored, community-based intervention among older Latino/as. Findings emphasize the need for future health services research, including public health strategies that address the unique cultural and socioeconomic needs of this population.

